# Autophagy in cancers including brain tumors: role of MicroRNAs

**DOI:** 10.1186/s12964-020-00587-w

**Published:** 2020-06-09

**Authors:** Mohammad Hossein Pourhanifeh, Maryam Mahjoubin-Tehran, Mohammad Reza Karimzadeh, Hamid Reza Mirzaei, Zahra Sadat Razavi, Amirhossein Sahebkar, Nayyerehsadat Hosseini, Hamed Mirzaei, Michael R. Hamblin

**Affiliations:** 1 Halal Research Center of IRI, FDA, Tehran, Iran; 2 Student Research Committee, Mashhad University of Medical Sciences, Mashhad, Iran; 3 Department of Medical Biotechnology, Faculty of Medicine, Mashhad University of Medical Sciences, Mashhad, Iran; 4 Department of Medical Genetics, School of Medicine, Bam University of Medical Sciences, Bam, Iran; 5 Department of Medical Immunology, School of Medicine, Tehran University of Medical Sciences, Tehran, Iran; 6 Student Research Committee, Kashan University of Medical Sciences, Kashan, Iran; 7 School of Medicine, Kashan University of Medical Sciences, Kashan, Iran; 8 Neurogenic Inflammation Research Center, Mashhad University of Medical Sciences, Mashhad, Iran; 9 School of Pharmacy, Mashhad University of Medical Sciences, Mashhad, Iran; 10 Medical Genetics Research Center, Department of Medical Genetics, Faculty of Medicine, Mashhad University of Medical Sciences, Mashhad, Iran; 11 Research Center for Biochemistry and Nutrition in Metabolic Diseases, Institute for Basic Sciences, Kashan University of Medical Sciences, Kashan, Iran; 12 Wellman Center for Photomedicine, Massachusetts General Hospital, Harvard Medical School, 40 Blossom Street, Boston, MA 02114 USA

**Keywords:** Brain tumors, Autophagy, MicroRNAs

## Abstract

Autophagy has a crucial role in many cancers, including brain tumors. Several types of endogenous molecules (e.g. microRNAs, AKT, PTEN, p53, EGFR, and NF1) can modulate the process of autophagy. Recently miRNAs (small non-coding RNAs) have been found to play a vital role in the regulation of different cellular and molecular processes, such as autophagy. Deregulation of these molecules is associated with the development and progression of different pathological conditions, including brain tumors. It was found that miRNAs are epigenetic regulators, which influence the level of proteins coded by the targeted mRNAs with any modification of the genetic sequences. It has been revealed that various miRNAs (e.g., miR-7-1-3p, miR-340, miR-17, miR-30a, miR-224-3p, and miR-93), as epigenetic regulators, can modulate autophagy pathways within brain tumors. A deeper understanding of the underlying molecular targets of miRNAs, and their function in autophagy pathways could contribute to the development of new treatment methods for patients with brain tumors. In this review, we summarize the various miRNAs, which are involved in regulating autophagy in brain tumors. Moreover, we highlight the role of miRNAs in autophagy-related pathways in different cancers.

Video abstract

Video abstract

## Background

Brain tumors arise from a wide variety of cell types, which give rise to tumors with different degrees of malignancy and invasiveness, and can afflict both adults and children [1, 2]. Despite the low incidence of these tumors, they are a leading cause of cancer-associated mortality and morbidity, particularly in young adults and children, where they account for about 20 and 30% of cancer deaths, respectively. Metastasis to the central nervous system (CNS) is also responsible for deaths in patients with other types of malignancies. In 2015, CNS tumors were estimated to represent 2.6% of cancer-related deaths as well as 1.4% of newly diagnosed cancers [3–5].

Because surgical brain tumor resection only leads to survival of a few months (median of 3 months), many studies have been done to improve the effectiveness of treatment, because complete tumor removal is usually impossible [6, 7]. The surgical elimination of the tumor is dependent on the glioma sub-type and its location within the brain [6]. However, although additional chemotherapy and radiotherapy can prolong median survival up to more than 1 year, tumor cells still develop resistance mechanisms to these therapies [8–10]. Identifying the detailed molecular mechanisms involved in tumor progression could reveal novel approaches to developing more effective therapies.

Several studies have revealed the contribution of autophagy in the pathogenesis of brain tumors [11, 12]. Autophagy is known to a well-conserved cellular pathway designed for recycling and degrading damaged or denatured proteins, together with long-lived or short-lived intracellular organelles [13, 14]. The autophagy process can be categorized into 3 sub-types called, micro-autophagy, macroautophagy, and chaperone-mediated autophagy [15]. Autophagy plays important roles in many biological functions ranging from embryonic development to cellular survival [16]. Dysfunction of the autophagic process has been correlated with a wide range of age-related diseases, such as CNS-related disorders and cancers [17]. In recent years, autophagy has begun to be investigated as a therapeutic target in several malignancies, such as breast cancer [18] and melanoma [19, 20]. There are currently a few anticancer treatments which act on autophagy pathways. Fortunately, compounds that directly or indirectly modulate autophagy are currently being studied in the context of phase I and phase II clinical trials [21]. Additionally, the regulation of autophagy has also been identified as an approach to the treatment of brain tumors in both children and adults [22–25].

Nevertheless, the precise role of autophagy in pediatric CNS tumors is not completely understood, which highlights the need to reveal additional details of autophagy-related processes to assess whether the contribution of autophagy inhibition to cancer therapy is a valid approach [26]. It has been shown that various cellular and molecular mechanisms are associated with autophagy-related processes in brain tumors and also in other different cancers. Along with genetic mechanisms, epigenetic mechanisms (e.g., miRNA networks) play major roles in the regulation of autophagy-related processes.

MicroRNAs (miRNAs) are short non-coding RNAs that bind to the 3′ untranslated regions of messenger RNAs (mRNAs) [27]. In fact, miRNAs can modulate the expression of more than 50% of each gene because each individual miRNA is able to target several different mRNAs [28, 29]. Therefore, the deregulated expression of miRNAs is likely to be related to the pathogenesis of many malignancies, such as brain tumors [30]. A variety of miRNAs are able to modulate autophagy, and its related mechanisms in various cancers including brain tumors [31–35]. Taken together, many reports suggest that a better understanding of the underlying molecular mediators (i.e. miRNAs), and their functions in autophagy pathways, could contribute to the discovery and advancement of novel treatment approaches for patients with brain tumors. Some reviews explained the role of miRNAs and autophagy in generally cancers [36, 37]. However until now, their specific roles in brain tumors have not been described. Herein, for first time, we summarize the various miRNAs which are involved in regulating autophagy in brain tumors. Moreover, we highlight the contribution of miRNAs to autophagy-related mechanisms in different cancers.

## Autophagy mechanisms

Autophagy (literally self-eating) is the conserved, regulated mechanism for an orderly degradation and recycling of various cellular elements that are damaged or unnecessary [38]. It is known that autophagy can be induced in mammalian cells by different factors, such as oxidative stress that leads to endoplasmic reticulum (ER) stress. During ER stress, autophagy serves as an essential protective response [39]. Autophagy was first fully investigated in yeast cells, and the terminolgy “autophagy genes, ATG” was agreed upon to describe the proteins involved. The two main regulatory pathways of the autophagy machinery include the ATG5/7-independent and ATG5/7-dependent pathways [40, 41].

According to the literature, the Unc-51-like kinase (ULK) complex containing several proteins, such as ATG101, ATG13, FIP200 (FAK-family interacting protein of 200 kDa), as well as ULK1/2 (mammalian ortholog of yeast ATG1) initiates conventional ATG5/7-dependent autophagy [42, 43]. Under non-stressed conditions, ULK1/2 is phosphorylated by the mammalian target of the rapamycin complex 1 (mTORC1) resulting in inactivation of the ULK complex [44]. On the other hand, the nutrient-sensitive mTORC1 is inhibited under nutrient-limiting conditions, and the ULK complex subsequently remains non-phosphorylated, and is therefore activated [45]. After activation, translocation of the ULK complex to the phagophore, has been shown to occur. After this, the class-III phosphatidylinositol 3-kinase (PI3K) complex containing VPS34 (phosphatidylinositol 3-kinase Vps34), VPS15, Beclin1, as well as ATG14 proteins, becomes activated [46]. This results in the formation of the mature autophagosome, after phagophore closure and extension. Two different ubiquitin-like conjugation systems, microtubule-related protein 1 light chain 3 (LC3) and the ATG5-ATG12 system, are the key modulators of the elongation and closure of the autophagosomal membrane [47–49]. In addition, ATG7 (E1-like enzyme) can activate ATG12, which is then transported into the ATG10 (E2-like enzyme) to eventually conjugate with ATG5 in the ATG5-ATG12 pathway. The non-covalent interaction of the ATG5-ATG12 complex with ATG16L leads to the formation of a large multimeric (E3-like) complex. This tripartite complex is capable of conjugating LC3 to phosphatidyl-ethanolamine (PE) to produce a LC3-PE conjugate (which is called LC3-II). LC3-II is then loaded into the phagophore [50–52]. In order to monitor the progression of autophagy, LC3-II protein is frequently employed as a biomarker, since it is localized to both the outer and inner membranes of autophagosomes and phagophores [53, 54].

**Fig. 1:**
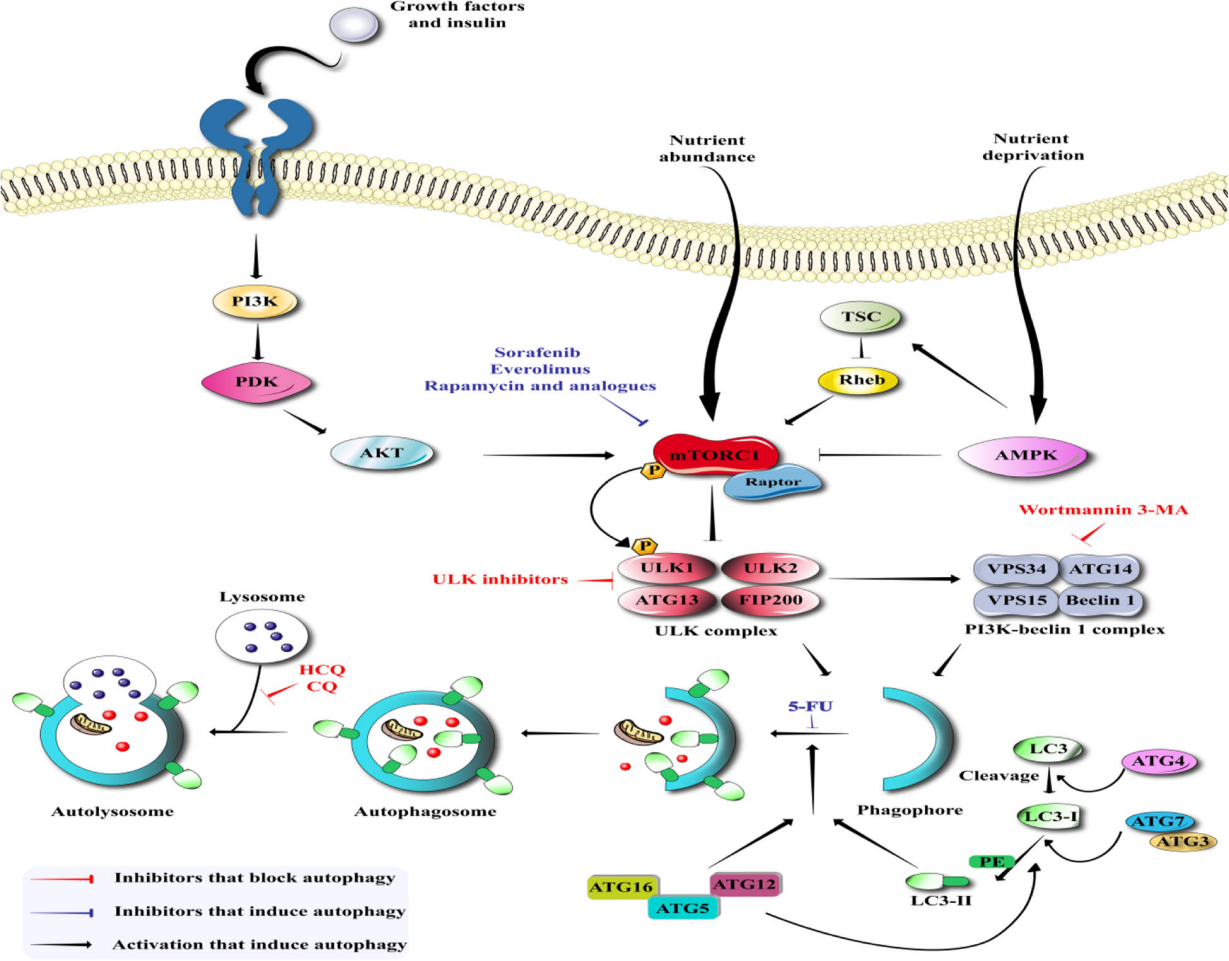
Autophagy mechanisms. Autophagy includes five steps: initiation, elongation, maturation, fusion and degradation. Various inhibitors can affect on these processes

Lysosomes, which are the degrading machinery in autophagy, are related to MTORC1 activation via the Rag/RRAG GTPase pathways. It has been shown that a MTORC1 inhibitor could suppress lysosomal degradation and increase lysosomal permeability [55]. The fusion of lysosomes with autophagosomes is the last step in the autophagy degradation cascade, which is triggered via three different sets of protein families: (a) RabGTPases (Rab7 protein) [56, 57]; (b) HOPS (homotypic fusion & the protein sorting tethering complex); and (c) the SNAREs (soluble N-ethylmaleimide-sensitive agent attachment protein receptors) [58–60]. Therefore, 3 distinct SNARE proteins; the vesicle-associated membrane protein 8 (VAMP8); synaptosomal-associated protein 29 (SNAP29); and syntaxin 17 (STX17) can all induce lysosome-autophagosome fusion [61, 62] (Fig. 1).

The mechanism of ATG5/7-independent autophagy has been discussed by Nishida and colleagues (2009) [43]. It was called “alternative autophagy” because ATG7 as well as ATG5 had been thought to be essential for autophagy [43]. The important finding in their study was that etoposide treatment of ATG5-deficient mouse embryonic fibroblasts (MEFs) triggered autophagy to a similar degree as in the wild type ATG5-expressing cells. Additionally, researchers found that PI3K, beclin1, and ULK1 complex played an important role, just as they do in conventional autophagy. Moreover, it was also found that silencing of the ATG5-ATG12 pathway had no effect on alternative autophagy, and that the conventional lipidation of LC3 was carried out by Rab9 activity to allow phagophore extension [43]. Rab9, which usually induces protein transport from the late endosomes to the *trans*-Golgi membrane, has been suggested to carry out phagophore closure and extension in the alternative autophagy pathway. This was the same process as carried out by ATG5/ATG7/LC3 in the conventional autophagy pathway [63–65]. There are multiple sources of phagophores in the ATG5/7-dependent autophagy pathway, but in the alternative autophagy pathway, the *trans*-Golgi cisternae seem to be the origin of the membranes [43, 66, 67].

**Fig. 2:**
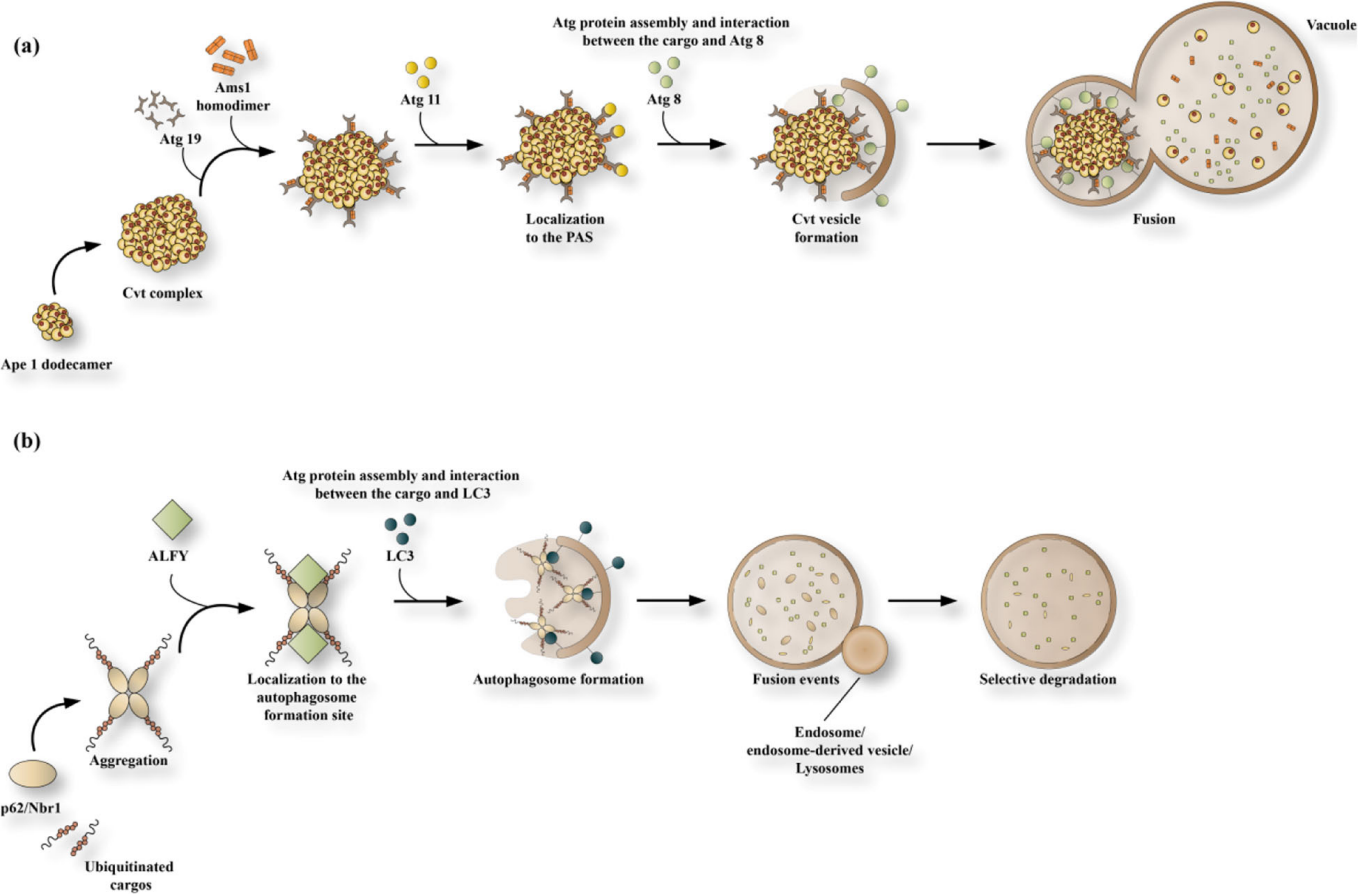
Mechanisms for selective autophagy. **a** Targeting pathway from the cytoplasm to the vacuole (Cvt). Ape1 is generated as a cytoplasmic precursor protein alongside a propeptide. The molecule will rapidly oligomerize into dodecamers. These dodecamers will link to each other to create higher-order composites. ATG19 as an autophagy receptor directly attaches to this complex and leads to another Cvt pathway cargo named Ams1 resulting in the formation of the Cvt complex and ATG19 interaction with an autophagy adaptor ATG11. The Cvt complex is transported to the location wherein the double-membrane vesicle will be created. ATG11 binds the ATG proteins required to generate Cvt vesicles. However, ATG19 direct binding to ATG8 allows unique sequestration of the Cvt complex into vesicles. **b** Scheme for p62 as well as NBR1 acting as autophagy receptors along with the ubiquitinated cargos. Furthermore, P62 and also NBR1 bind to the ubiquitinated cargo through their ubiquitin-associated (UBA) domain. This interaction initiates aggregate generation via oligomerization of p62 through its Bem1p (PB1) and Phox domains. p62 interacts with autophagy-linked FYVE protein (ALFY) to activate ATG5 and bind PI3P, as well as direct binding to LC3. These mechanisms seem to control and activate the ATG function along with the ubiquitinated cargos, and specifically sequester them inside autophagosomes, similar to the Cvt pathway

Studies have shown that autophagy is a critical quality control function in cellular processes. Autophagy at baseline levels operates to sustain cellular hemostasis. Some organelles undergo selective turnover through the autophagy mechanism. Various autophagy pathways can be distinguished by categorizing the contents of the autophagosomes. These pathways include, lipid droplets (lipophagy), ER (reticulophagy or ERphagy), secretory granules (zymophagy), mitochondria (mitophagy), and even some parts of the nucleus (nucleophagy). Furthermore, proteins that are prone to aggregation (aggrephagy), ribosomes (ribophagy), and pathogens (xenophagy), can be specifically targeted and degraded by autophagic processes [68]. Some types of autophagy function as cellular quality control mechanisms. These might be able to distinguish their substrates, including dysfunctional mitochondria or protein aggregates from their fully functional counterparts. Cargo selection, as well as autophagy regulation, still have mechanistic aspects that remain to be discovered, and this has been a focus of intense research interest in recent years. Recently a genome-wide small interfering RNA screening study was carried out to identify the various mammalian genes that are necessary for selective autophagy. This study discovered 141 candidate genes, of which 96 of them were required for Parkin-mediated mitophagy [69]. It appears that these pathways involve particular cargo-recognizing autophagy receptors that link the autophagic membranes to the cargo. These receptors may also interact with specific molecular adaptors, which act as scaffolding proteins. These proteins may help to connect the cargo receptor complex to the core ATG machinery. This connection allows selective sequestration of the substrate. There are other types of autophagy that are based on similar core molecular machinery to the non-selective (starvation-induced) bulk autophagy. On the contrary, specific molecular adapters or autophagy receptors are not necessary for the non-selective autophagy pathway. Autophagy receptors have been proposed as being capable of directing interaction with ATG8/LC3 family members, as well as the autophagosome cargo via specific (WxxL) sequences [70]. These are usually referred to as LC3 recognition sequences (LRS) or LC3-interacting region (LIR) motifs [71, 72]. By comparing the LIR domains among 20 different autophagy receptors, studies found that the LIR consensus recognition motif comprised a sequence of 8 amino acids. This sequence can be written down as D/E-D/E-D/E-W/F/Y-X-X-L/I/V. This is not, however, an essential condition, because at least one acidic residue upstream of the W-site exists. On the other hand, the terminal L-site contained a hydrophobic aminoacid residue, L, V, or I [73]. It was found that the LIR motifs of numerous autophagy receptors could all interact with both GABARAP and LC3 family members in vitro. But whether this interaction actually occurs under physiological conditions should be elucidated in most cases. It may be the case that all the LIR-containing proteins are not necessarily autophagy-cargo receptors. For example, there are a number of LIR proteins, such as ATG4B as well as ATG3, which could function in the autophagic membrane to produce autophagosomes [74, 75]. Other proteins, such as the coiled-coil domain-containing protein 1 (FYCO1) and FYVE can interact with LC3 to promote autophagosome maturation [76]. Other proteins, including Dishevelled, act as adaptors in the Wnt signaling pathway, and may exploit an LIR motif for their degradation [77]. These adaptor proteins have not yet been completely described, but they appear to interact with autophagy receptors and act as scaffold proteins for the assembly of the ATG machinery. This allows the production of autophagosomes that surround the cargo that is required to be degraded. ATG11 and ALFY are examples of these autophagy adaptors [78, 79]. The cytoplasm-to-vacuole targeting (Cvt) pathway mediates the transportation of some vacuolar hydrolases, such as amino-peptidase 4 (Ape4), *α*-mannosidase (Ams1), aminopeptidase 1 (Ape1), and Ty1 transposon (in yeast) into the vacuole [80, 81]. Ape1 is generated from a cytosolic precursor (prApe1), which undergoes multimerization into higher order Ape1 oligomers. Ams1, Ty1, and Ape4 then associate with the Ape1 oligomer to generate the fully assembled Cvt complex, which is sequestered in a small autophagosome-like vesicle. Sequestering the Cvt complex inside the Cvt vesicle is a multistage process, requiring the autophagy receptor ATG19 that promotes ATG8 binding to PAS, and to adaptor protein ATG11 (Fig. 2a) [82]. ATG11 functions as a scaffold protein by controlling the ATG9 reservoir, and allowing the Cvt complex to bind to PAS in an actin-dependent manner, and consequently forming the ATG1/ULK complex [83]. ATG20, ATG21, and ATG24 are PI3P-binding proteins, which have been found to be necessary for the Cvt function, however, the exact functions of these proteins is not yet clear. Surprisingly, over-expression of ATG11 led to greater ATG9 and ATG8 binding to PAS and more Cvt vesicles [84]. These results suggest that the level of ATG11 can selectively control the autophagy rate. ATG11 can also control the size of the cargo-loaded autophagosomes in yeast [85]. Some studies have shown that ATG11 can contribute to other types of selective autophagy, including pexophagy and mitophagy. The individual autophagy receptors participating in different ATG11-dependent types of selective autophagy vary, because ATG32 is necessary for mitophagy, while ATG30 is required for pexophagy [86, 87]. Therefore, these receptors possess an ATG8-binding LIR motif similar to ATG19, that governs the interaction with ATG11. It seems that mammalian cells do not have the ATG11 homologue. More investigations are required to define the molecular mechanisms that govern the sequestering as well as targeting the various cargoes to be broken down by autophagy in different eukaryotes. The Cvt pathway machinery appears to be similar to the mammalian autophagy, named aggrephagy. This entails the degradation of misfolded and unwanted proteins via assembling them into ubiquitinated aggregates. Thus, aggregation of the substrates (prApe1 or misfolded proteins) is essential before sequestering them into the Cvt vesicles or autophagosomes [88]. Aggregate-containing autophagosomes, similar to Cvt vesicles, seem to not contain any cytosolic elements, demonstrating the well-controlled expansion of the vesicle membrane surrounding its cargo [78]. Besides, aggrephagy is dependent on the unique functions of proteins that select the substrate [89]. The p62 autophagy receptor and adjacent BRCA1 gene (NBR1) bind the ubiquitinated protein aggregates via an ubiquitin-associated (UBA) domain, as well as LC3, through their LIR motifs. This process ensures the selective autophagic breakdown of the ubiquitinated proteins (Fig. 2b) [90].

p62 and NBR1 also include a Bem1p (PB1) domain and an N-terminal Phox by which they can be oligomerized, or interact with PB1-containing binding partners [73]. Moreover, p62 has been implicated as a cargo receptor for protein aggregates in the autophagic breakdown of additional ubiquitinated substrates. These substrates include viral capsid proteins, intracellular bacteria, peroxisomes, midbody remnants formed after cytokinesis, bactericidal precursor proteins, and damaged mitochondria [91]. It has been recently found that the PB1 domain was necessary for p62 to restrict the autophagosome genesis site in the vicinity of the ER. It was suggested that PB1 may target the ubiquitinated cargos to the location of autophagosome formation, or alternatively to improve the assembly of ATG complexes at the site [91].

## MicroRNA biogenesis

MicroRNAs are non-coding single-stranded RNA molecules with a length of approximately 17–25 nt. These molecules modulate biological processes by posttranscriptional gene silencing [92]. Necrotic cells are able to release the miRNAs as naked oligonucleotides, or else they are secreted contained in extracellular vesicles. Furthermore, researchers have detected circulating miRNAs (c-miRNAs) in many body fluids, such as *cerebrospinal fluid* (*CSF*)*.* It has been proposed that these c-miRNAs play a role in intercellular communication, and thus can possibly affect various cellular processes at a molecular level, such as cell growth and invasiveness, and can also affect drug resistance in the recipient target cells [93, 94].

**Fig. 3:**
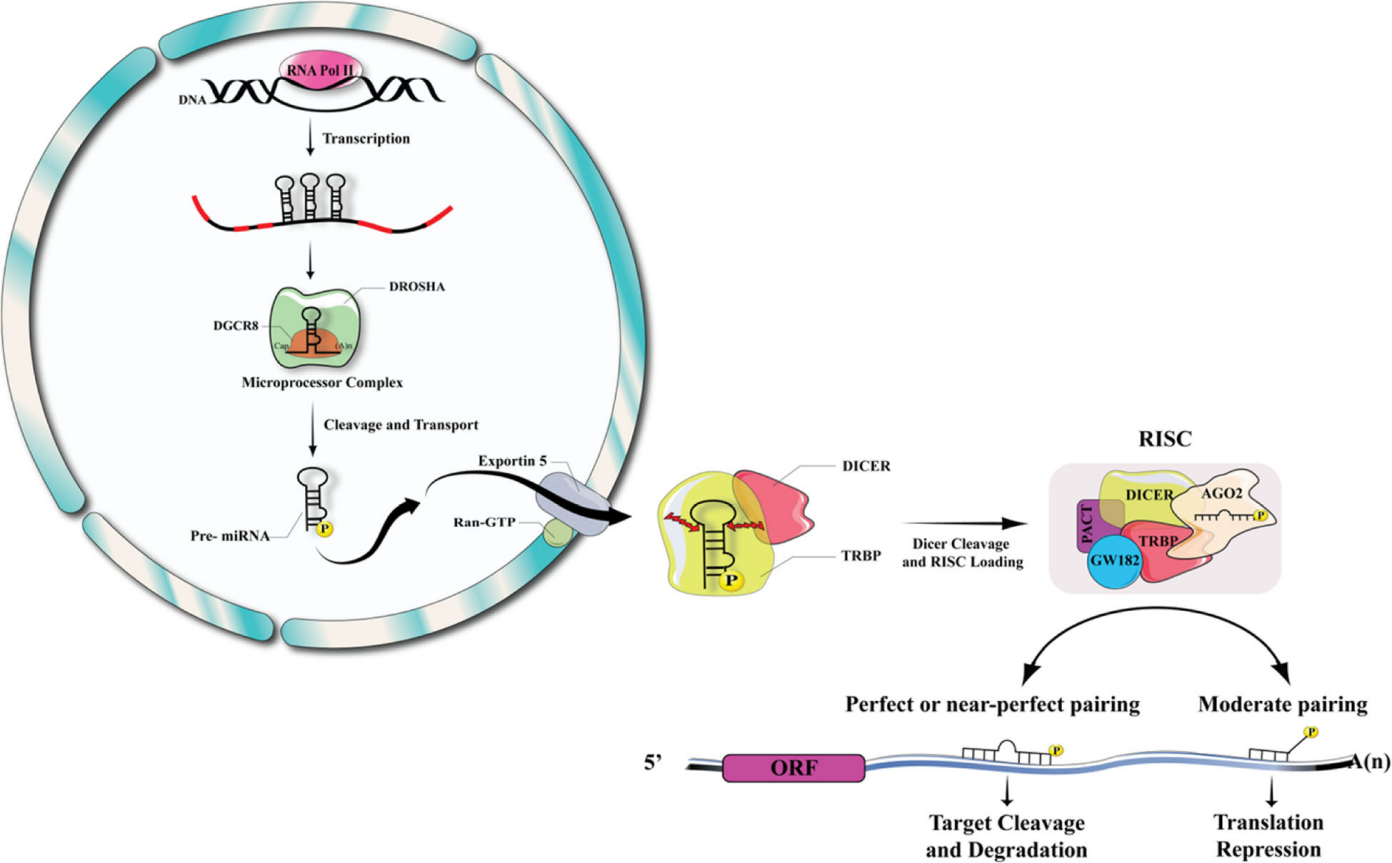
MicroRNA processing and function. In order to produce hairpin-structured pre-miRNAs, a core ribonuclease complex, such as Drosha or the respective modulatory sub-unit DGCR8 is used to process them in the nucleus. Following cleavage, these hairpin-structured premiRNAs are transferred from the nucleus into the cytoplasm. Moreover, further cleavage of the pre-miRNA hairpin structure in the cytoplasm is carried out by DICER protein, resulting in the formation of long miRNA duplexes. These duplexes are loaded into the RNA-induced silencing complex (RISC). In addition, Argonaute (AGO) proteins are essential elements of the RISC that direct mature single-stranded miRNAs to their target mRNAs. However, the destiny of the targeted mRNA is governed by the interrelationship between the miRNA response elements (MRE) and the mature miRNA seed sequences. Therefore, base-pairing of the target mRNA to the guide miRNA leads to its endonuclease-mediated cleavage in a slicer-dependent manner

Long primary pri-miRNAs or miRNAs are usually transcribed from miRNA genes by RNA polymerase II [95]. Pri-miRNAs may occasionally produce several different functional miRNAs [95]. In order to produce hairpin-structured pre-miRNAs, a core ribonuclease complex, such as Drosha or the respective modulatory sub-unit DGCR8 is used to process them in the nucleus. Following cleavage, these hairpin-structured premiRNAs are transferred from the nucleus into the cytoplasm. Moreover, further cleavage of the pre-miRNA hairpin structure in the cytoplasm is carried out by DICER protein, resulting in the formation of long miRNA duplexes. These duplexes are loaded into the RNA-induced silencing complex (RISC). In addition, Argonaute (AGO) proteins are essential elements of the RISC that direct mature single-stranded miRNAs to their target mRNAs. However, the destiny of the targeted mRNA is governed by the interrelationship between the miRNA response elements (MRE) and the mature miRNA seed sequences. Therefore, base-pairing of the target mRNA to the guide miRNA leads to its endonuclease-mediated cleavage in a slicer-dependent manner. The degradation process can be proceeded by miRNA-mediated deadenylation and/or de-capping of the target mRNA, while the translation machinery may be blocked by partial complementary binding (Fig. 3) [96, 97].

## Regulation of autophagy by microRNAs in cancer

Over the past decade, it has been found that miRNAs are able to control a number of ATGs (and their respective modulators) at various steps of the autophagy process, including vesicle nucleation, induction, retrieval, fusion, and finally vesicle elongation (Fig. 3) [98]. The first step in the induction of autophagy is triggered by ULK complex activation. This complex includes the components, ULK1/2, FIP200, and FIP200 [98, 99]. Moreover, ULK1 protein kinase has been considered to be the main initiator of the autophagic process. In nutrient-rich conditions, mTOR is able to phosphorylate the mammalian ATG13 (mATG13) and ULK1 that together prevent the activation of ULK1 kinase. However, under starvation conditions, mTOR is inactivated which then allows ULK1 to phosphorylate FIP200 and mATG13 as well as itself. This leads to engagement of ATG complexes, like class-III phosphatidylinositol 3-kinase (PI3KCIII) to initiate autophagy. In addition, the miR-290–295 cluster was shown to down-regulate ULK1 levels, so that ATG7 inhibited autophagic cell death caused by glucose starvation [100]. Leucine deprivation also repressed expression of miR-106b and miR-20a through repression of the transcription factor c-Myc. Transfection of miR-20a or miR-106b mimics could hamper the leucine deprivation mediated autophagy in C2C12 myoblasts. This mechanistic investigation suggested the probable targeting of ULK1 by miR-106b and miR-20a, as well as directly preventing its expression [101]. A simple chalcone-type flavonoid compound, called isoliquiritigenin can be isolated from liquorice compounds. This flavonoid mediated cell cycle arrest, chemo-sensitization, as well as autophagy in multidrug resistant MCF7 cells. This mechanic investigation suggested that miR-25 was a key target of soliquiritigenin. Moreover, miR-25 suppression caused autophagic cell death via direct ULK1 over-expression [102].

It was reported that miR-126 was down-regulated in malignant mesothelioma tissue, and also that its over-expression inhibited cancer cell growth, probably because of its impact on their metabolism. miR-126 also led to energy deprivation, reduced glucose uptake, and inhibited IRS1, resulting in ULK1 activation [103]. Furthermore, miR-126 also affected other metabolism-associated proteins, including acetyl-CoA-citrate and pyruvate dehydrogenase kinase. These metabolic changes induced by miR-126 resulted in the suppression of tumor growth and activation of autophagy both in vitro and in vivo [103].

It was recently reported that ULK2, which is another up-stream autophagy initiator, is a direct target of miR-885-3p. Therefore, miR-885-3p may be involved in autophagy modulation [104]. Vesicle nucleation was induced by activation of the class-III PI3K/Beclin-1 complex. This complex has numerous binding partners, including hVPS34, UV-irradiation resistance-associated gene (UVRAG), Bax-interacting factor-1 (BIF-1), ATG14L, and Rubicon. Several miRNAs, including miR-376b, miR-30a/b, miR-17-5p, and miR-216a can all hamper Beclin-1 expression, and inhibit vesicle nucleation [105–108]. In one study Huang et al. showed that miR-519a could directly target Beclin-1 [109]. Furthermore, it was shown that miR-374a (as well as miR-630) could inhibit the interaction of UVRAG with Beclin-1, resulting in autophagy activation. ATG14 is a vital component of the class III PI3K/Beclin-1 complex in the nucleation of autophagosomal membranes. ATG14 has also been recognized as a miR-195 target [110]. Moreover, RAB5A a small GTPase, which interacts with Beclin-1 and hVPS34 is able to mediate autophagosome formation. In addition, RAB5A can also be targeted by miRNA-101 to inhibit autophagy, showing that miR-101 can regulate autophagy at the vesicle nucleation stage [111, 112]. Thirdly, two different ubiquitin-like conjugation mechanisms can act to elongate the vesicle: one is the ATG8-phosphatidyl ethanolamine mechanism and the other is ATG12-ATG5-ATG16L. Several proteins are involved in this process, including ATG7, ATG10, ATG4, ATG5, ATG3, ATG12, ATG16L, as well as microtubule-related protein 1 light chain 3 (LC3). MiR-376b and miR-101 can negatively modulate ATG4C and ATG4D expression [107, 112]. MiR-376a was found to have a similar seed sequence and similar targets to miR-376b, including Beclin-1 [113] and ATG4C (90).

In hepatocellular carcinoma (HCC) cells, the conversion of LC3-I into LC3-II is inhibited by miR-375 via targeting of ATG7 [114]. Besides, miR-17 could also decrease the expression of ATG7 in glioblastoma cell lines [115]. Furthermore, RAB5A participates in the conjugation of ATG5 to ATG12 [112]. Thus, miR-101 by targeting RAB5A, could have an impact on both the nucleation and elongation of the vesicles. In addition, miR-204 was able to modulate autophagy in renal clear cell carcinoma (RCC) by modulating LC3B [116]. MiR142-3p, miR-106B, and miR-93 can all stimulate autophagy by targeting ATG16L [117, 118]. Whereas, miR-519a, miR-130a, miR-30a/c, miR-885-3p, miR-630, miR-181a, and miR-374a, can repress autophagy by targeting ATG5-ATG12 conjugation [119, 120]. The fusion and retrieval process of autophagosomes could be modulated by targeting UVRAG, ATG9, ATG18, as well as ATG2. Many different miRNAs can participate in this final stage of autophagy. Moreover, ATG2B has been established as one of the direct targets of miR-130a [121]. Thus, MiR-34 suppresses autophagy via decreasing ATG9 expression in mammalian cells [122]. Jegga et al. investigated the transcriptional as well as the post-transcriptional modulation of ATGs mediated by miRNAs. They showed that miR-130, 124, 98, 142, and 204 were all involved in the modulation of autophagy-lysosomal pathway genes. UVRAG is also one of the key molecules in the fusion process. The miRNAs that target UVRAG including miR-374a as well as miR-630, may participate in the modulation of autophagosome–lysosome fusion [109]. There are also some other miRNAs that could be involved in autophagy modulation. BCL-2 binds to Beclin-1, and consequently inhibits Beclin-1-dependent autophagy. MiR-182, miR-34a, miR-210, miR-21, and miR-205 can target BCL-2, and are likely to modulate autophagy via the BCL-2/Beclin-1-PI3KIII pathways [123–126]. The p62 protein, called sequestosome 1 (SQSTM1), is a selective substrate for autophagy, and also acts a scaffold in the autophagosome. The miR-17/20/93/106 has a common AAGUGC seed motif, and can directly modulate the expression of p62, suggesting a possible role in autophagy modulation [126]. The hypoxia-induced miR-155 can promote autophagy by targeting numerous genes in mTOR signaling pathways, such as RICTOR, RPS6KB2, as well as RHEB [127]. Furthermore, miR-100 is able to enhance autophagy in hepatocellular carcinoma cells via targeting IGF-1R as well as mTOR [128]. Histone deacetylases (HDACs) and histone acetyltransferases (HATs) have a key role in epigenetic regulation by affecting protein acetylation. MiR-9 and miR-206 can modulate HDAC and HAT expression in Waldenstrom macroglobulinemia (WM) cells, leading to autophagy dependent cell death [129]. HDAC6 is a prominent cytoplasmic deacetylase, that targets heat shock protein 90, cortactin, and tubulin. Thus, HDAC6 can modulate cell motility, adhesion, and chaperone function [130]. It has been shown that HDAC6 has a role in carcinogenic transformation and may regulate the epithelial-mesenchymal transition (EMT) in various types of cancer via modulation of major cellular components. Many pieces of evidence suggest that HDAC6 expression is correlated with tumor aggressiveness, anchorage-independent proliferation, and oncogenic transformation [131, 132]. Studies have shown that HDAC6 has a role in the clearance of aggresomes. These studies also pointed out a functional connection between autophagy and HDAC6 [133]. Another study showed that transfection with miR-221 mimics could inhibit HDAC6 expression in pancreatic cancer cells compared to negative controls [133]. In pancreatic cancer cells, the suppression of HDAC6 could mediate autophagy. Down-regulation of miR-221 expression via increasing HDAC6 function could play an oncogenic role in suppressing autophagy and apoptosis in pancreatic cancer cells [133].

Long non-coding RNAs are another member of the class of non-coding RNAs that could have a crucial role in cancer pathogenesis [134]. It is thought that lncRNAs exert their modulating roles via sponging of miRNAs and proteins. The physiological and biological roles of autophagy-regulating lncRNAs in cancer have recently been appreciated. The expression of lncRNAs substantially affects the level of autophagy at various steps of carcinogenesis, and especially in advanced metastatic cancer [134]. It has been proposed that impaired expression of MALAT1 (metastasis associated lung adenocarcinoma transcript 1) regulates autophagy in different cancers such as RTB, HCC, glioma, and GC via modulation of miRNAs, miR-101, miR-124, miR-23b-3p, as well as miR-216b [135–138]. Autophagy and GAS5 expression were both decreased in breast cancer cells. Furthermore, the GAS5 expression levels in patient samples showed a negative correlation with tumor size, depth, TNM stage, as well as with poor clinical prognosis. Surprisingly, vector-induced GAS5 over-expression initiated autophagy, and also elevated p62, LC3, and ATG3 expression through sponging of miR-23a. This result could be helpful as a novel treatment for breast cancer via modulating the GAS5/miR-23a/ATG3 axis [139].

**Fig. 4:**
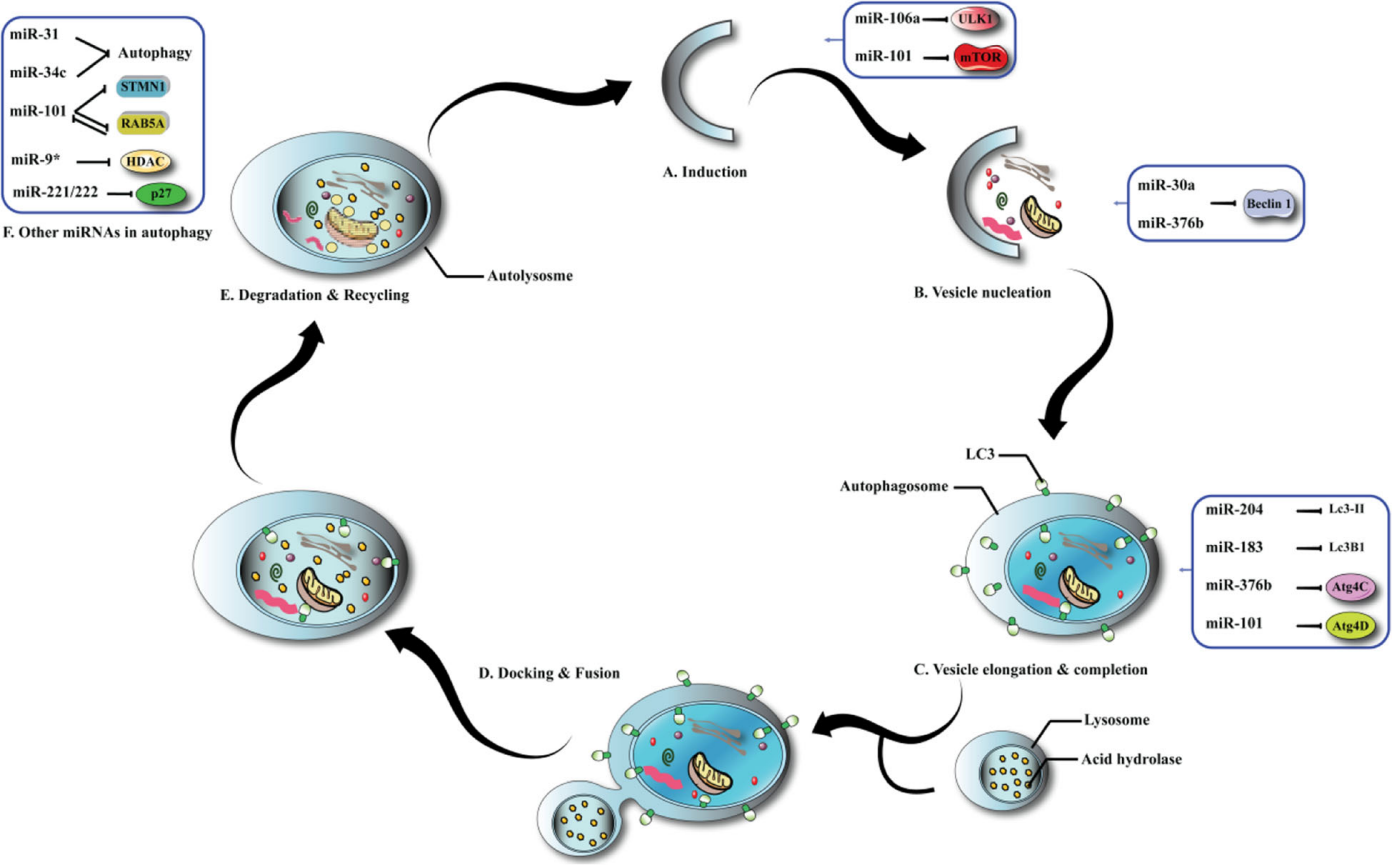
Various microRNAs involved in autophagy-related mechanisms. MiR-31, miR-34a miR-9 and miR-101 are able to affect on degradation and recycling. MiR-204, miR-183, 101, and miR-376b affect on Autophagosome

RNA editing involves discrete changes being made to specific nucleotide sequences within an existing RNA molecule. It has recently been shown that the process of A-to-I RNA editing can alter miRNA function [140]. For instance, compared to the wild-type miRNA, the edited miR-200b could increase the invasion and migration of cancer cells [141]. In another study, it was shown that the edited miR-379-5p, as opposed to the wild-type miR-379-5p that targets CD97, suppressed rapid cell proliferation and increased apoptosis in tumor cells in-vitro [142]. Table 1 and Fig. 4 lists various autophagy-related miRNAs that have been reported to be involved in cancer.

**Table 1:** Selected autophagy-associated miRNAs in cancer

Cancer	MicroRNA	Target (s)	Effect (s)	Cell line (s)	Ref
Melanoma	miR-290*-*295	ATG7, ULK1	Inhibit autophagy	R2L, B16F1	[100]
	miR-638	TP53INP2	Inhibit autophagy	SK-Mel-147, SK-Mel-28	[143]
Esophageal cancer	miR-193b	STMN1	Activate autophagy	KYSE450	[144]
	miR-634	BIRC5, XIAP, APIP, TFAM, OPA1, LAMP2	Inhibit autophagy	KYSE850	[145]
Squamous cell carcinoma	miR-374a	ATG5, UVRAG	Inhibit autophagy	JHU-029	[146]
	miR-630	UVRAG, ATG12	Inhibit autophagy	JHU-029	[146]
	miR-519a	BECN1, ATG16L1, ATG10	Inhibit autophagy	JHU-029	[146]
	miR-885*-*3p	AKT1, ULK2, ATG16L2, BCL-2	Inhibit autophagy	JHU-029	[147]
Colorectal cancer	miR-18a	hnRNPA1	–	HCT116, SW620	[148]
	miR-22	BTG1	Inhibit autophagy	RKO, SW620	[149]
	miR-93	ATG16L1	Inhibit autophagy	HCT116	[150]
	miR-106	ATG16L1	Inhibit autophagy	HCT116	[150]
	miR-183	UVRAG	Inhibit autophagy	HT29, HCT116	[151]
	miR-409*-*3p	BECN1	Inhibit autophagy	LovoOxa R	[152]
	miR-502	RAB1B	Inhibit autophagy	HCT116	[153]
Gastric cancer	miR-143	GABARAPL1	Inhibit autophagy	MKN28, AGS	[154]
	miR-181a	ATG5	Inhibit autophagy	SGC7901/CDDP	[155]
Lung cancer	miR-7	EGFR	Activate autophagy	A549, H1299	[156]
	miR-16	BCL-2	Inhibit autophagy	A549-T24	[157]
	miR-17*-*5p	BECN1	Inhibit autophagy	A549-T24	[105]
	miR-143	ATG2B	Inhibit autophagy	H1299	[158]
	miR-144	TIGAR	Activate autophagy	H460, A549	[159]
	miR-200b	ATG12	Inhibit autophagy	H1299/DTX, SPC-A1/DTX	[160]
	miR-216b	BECN1	Inhibit autophagy	Calu-3, A549	[161]
	miR-451	RAB14	–	A549, NCI-H520, SPC-A1	[162]
	miR-487b-5p	LAMP2	Inhibit autophagy	H1299, A549	[163]
Breast cancer	miR-25	ULK1	Inhibit autophagy	MCF-7	[102]
	miR-181a	ATG5	Inhibit autophagy	MCF-7	[119]
	miR-199A*-*5p	BECN1, DRAM1	Inhibit autophagy	MDA-MB-231, MCF-7	[164]
	miR-200c	UBQLN1	Activate autophagy	MDA-MB-231	[165]
	miR-372	SQSTM1	Inhibit autophagy	MCF10A, MCF-7	[166]
	miR-376b	ATG4C, BECN1	Inhibit autophagy	MCF-7	[113]
	miR-451a	–	Inhibit autophagy	LCC2, MCF-7	[167]
Ovarian cancer	miR-152	ATG14	Inhibit autophagy	SKOV3/DDP, A2780/CP70	[168]
	miR-373	RAB22A	Inhibit autophagy	SKOV3	[169]
Cervical cancer	miR-15a*/*16	RICTOR	Activate autophagy	HeLa	[170]
	miR-20a	ATG7	–	SiHa	[171]
	miR-155	RHEB, RPS6KB2, RICTOR	Activate autophagy	HeLa, NSE	[127]
	miR-224*-*3p	FIP200	Inhibit autophagy	SiHa, HeLa, C33A	[172]
Endometrial carcinoma	miR-218	HMGB1	Inhibit autophagy	RL95–2	[173]
Prostate cancer	miR-96	ATG7, mTOR	Inhibit autophagy	LAPC4, 22Rv1, LNCaP	[174]
	miR-124	PIM1	Inhibit autophagy	PC3, DU145	[175]
Liver cancer	miR-21	PTEN	Inhibit autophagy	HepG2, Huh7	[176]
	miR-101	EZH2	Inhibit autophagy	HepG2	[177]
	miR-199A*-*5p	ATG7	Inhibit autophagy	HepG2, Huh7	[178]
	miR-224	SMAD4	Inhibit autophagy	Hbx, Hep3B	[179]
	miR-375	ATG7	Inhibit autophagy	Hep3B, Huh7	[114]
	miR-376b	ATG4C, BECN1	Inhibit autophagy	Huh7	[113]
Pancreatic cancer	miR-23a	ATG12	Inhibit autophagy	BxPC3	[180]
	miR-216a	BECN1	Inhibit autophagy	PANC-1	[181]
Kidney cancer	miR-214	LC3B, LC3A	Inhibit autophagy	A498, 786-O, Caki-1	[182]
Thyroid cancer	miR-9*-*3p	ATG5	Inhibit autophagy	MZ-CRC-1, TT	[183]
Hepatocellular carcinoma	miR-17	PTENP1, PTEN	Activate autophagy	Mahlavu	[184]
	miR-19b	PTENP1, PTEN	Activate autophagy	Mahlavu	[184]
	miR-20a	PTENP1, PTEN	Activate autophagy	Mahlavu	[184]

## Autophagy and brain tumors: paving the way for the development of new drugs

### Alterations in autophagy in brain tumors

The poor response of malignant brain tumors to conventional therapies, many of which work by inducing apoptosis, makes it attractive to target autophagy as an alternative mechanism for triggering glioma cell death [185, 186]. Alterations or mutations that are commonly found in brain tumors, include *p53, PTEN, AKT, NF1 and EGFR*, and some of these are accepted to be implicated in the modulation of autophagy [185, 186]. Considering the frequencies of mutations in *EGFR, p53, PTEN, NF1,* and *PDGFR*, the Cancer Genome Atlas consortium categorized glioblastoma (GBM) tumors into four molecular sub-types, including neural, classical, mesenchymal, and proneural [187]. Researchers have also found differences in basal expression levels of the LC3 protein in xenografts of the GBM four subtypes, which were associated with differences in the susceptibility to autophagy. It has been proposed that combinational approaches targeting autophagy-lysosomal related mechanisms might result in improved GBM subtype-specific treatments. Furthermore, it has been shown that autophagy can be activated by some experimental glioma treatments. Although autophagy can increase the survival and resistance of tumor cells under some circumstances, autophagy is also able to exert cytostatic and/or cytotoxic effects in other therapeutic approaches. The particular role of autophagy in contributing to cell death or cell survival in different therapeutic approaches is yet to be fully explained, and a better understanding of these contrary findings is essential to design potential combination therapies [187].

**Fig. 5:**
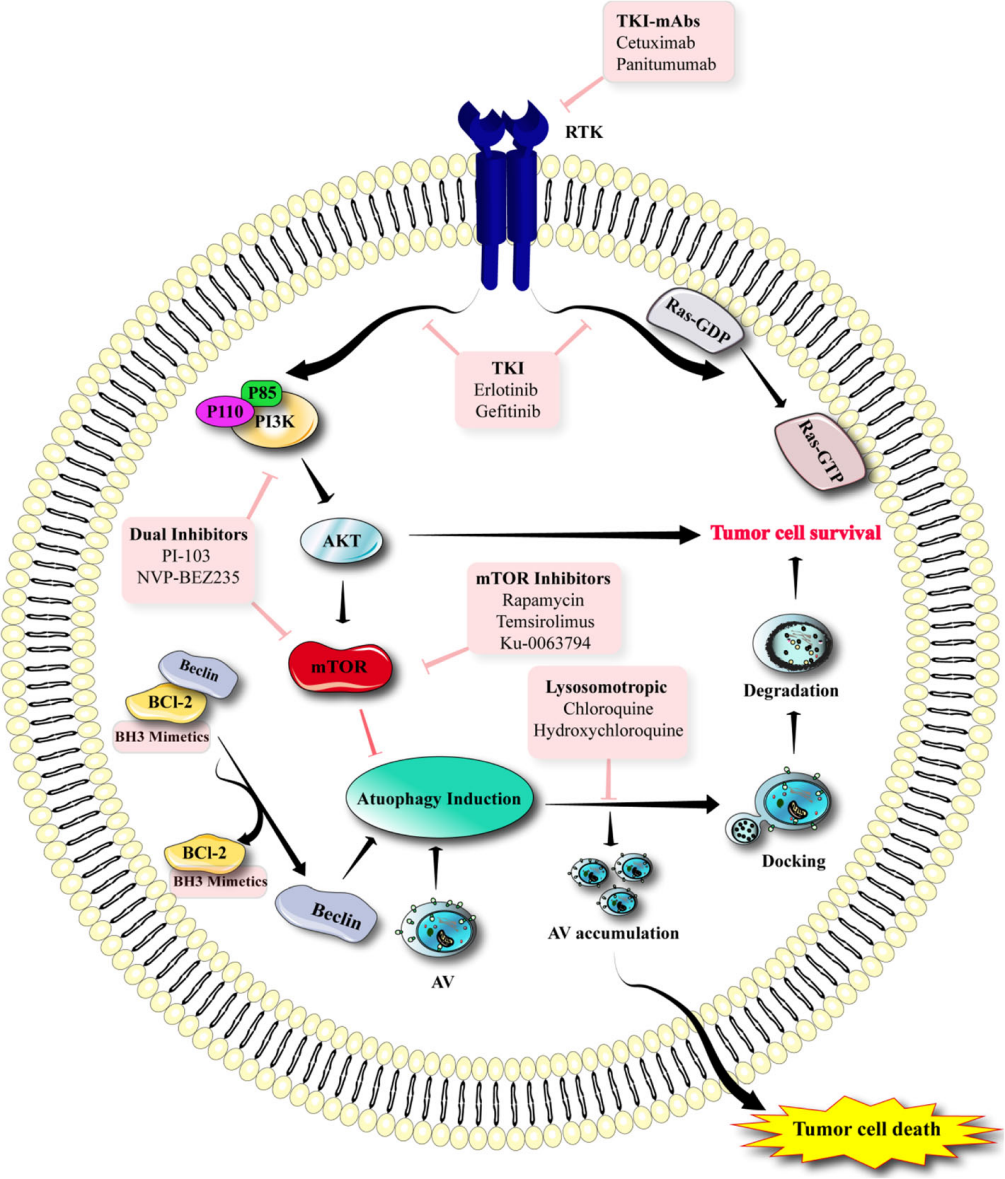
Autophagy in brain tumors and possible role of inhibitors in brain tumor treatment. Various inhibitors exert their effect on different targets in glioma cells. Dual inhibitors (PI-103 and NVP-BEZ235) inhibit mTOR. TKI inhibits RTK and PI3K

The most frequent genetic alterations/mutations found in gliomas are: hemizygous/homozygous deletion of NF-1 and PTEN; EGFR vIII mutant expression; and EGFR amplification [187]. The abnormal signaling resulting from such mutations interacts with PI3K-Akt-mTOR pathways that promotes chemo-resistance and survival in gliomas [188]. Thus, the idea to target receptor tyrosine kinases (RTKs) using small molecule inhibitors, or else with monoclonal antibodies has emerged as a favored therapeutic approach (Fig. 5).

### Development of therapeutic drugs

Disappointingly, the first clinical trials that were conducted with small molecule EGFR inhibitors, including gefitinib and erlotinib that had shown success for other tumor types, did not show any encouraging results for glioma treatment [189–191]. Likewise, monoclonal antibodies targeting EGFR (panitumumab and cetuximab) only exerted cytostatic effects on glioma cell lines [192, 193]. However, the variety of different types of RTK that are found in gliomas (combined with frequency of PTEN elimination) could explain the ineffectiveness of tyrosine kinase inhibitors (TKI) each of which only targets a single enzyme [194–196]. Moreover, preclinical investigations using selected mTOR and PI3K inhibitors, have shown only a moderate efficacy against gliomas. A phase-II trial using temsirolimus (CCI-779), a mTOR blocker, did not improve survival in patients with recurrent GBM [197]. However, PI-103, a dual functional mTOR and PI3K inhibitor, did show beneficial anti-proliferative effects in preclinical glioma models, by suppressing the Akt activation often found with mTOR blockers [198]. Treatments which targeted components of the RTK-PI3K-Akt-mTOR axis, increased the induction of autophagy with an overall cytoprotective effect. Therefore, a combination of inhibitors of late stage autophagy plus other compounds that actually induce autophagy may work together to increase the cytotoxicity in gliomas. Indeed, this concept was tested in one approach, which combined the lysosomotrophic agent chloroquine (CQ) that blocked the activity of lysosomal proteases, with the PI3K/mTOR/AKT inhibitors (AKT-1/2 and PI-103), resulting in the overall potentiation of glioma cell death [199]. Furthermore, NVP-BEZ235 (a dual mTOR and PI3K inhibitor currently being tested in clinical trials) induced autophagy in solid tumors, and synergistically acted in combination with CQ through increased apoptosis in glioma cells [200]. A combination of monensin or bafilomycin A1 (both late-stage autophagy inhibitors) with Ku-0063794 or PI-103 also promoted glioma cell death via apoptosis induction [200]. Also, experts in the field have proposed that inhibition of autophagy could act synergistically with erlotinib for induction of cytotoxicity in GBM cells [201]. In the case of a combination of autophagy inhibitors with the TKI, imatinib mesylate, the precise stage at which autophagy was suppressed led to different outcomes in gliomas. Early-stage inhibition of autophagy using small interfering RNAs targeting ATG5 or 3-methyladenine (3-MA) decreased imatinib cytotoxicity, whereas conversely suppression of late-phase autophagy using bafilomycin A1 increased cytotoxicity by inducing more apoptosis [202]. Therefore, the varying outcomes of autophagy suppression under different conditions may depend on the specific compound that targets different stages of autophagy, and on other factors that are not yet completely understood.

Although the combination of drugs which induce autophagy, with agents that inhibit the completion of autophagy, appear to be somewhat promising up to now, and some clinical trials are actually in progress, there are other treatments that can contribute to autophagy-associated glioma cell death. For example, adding an inducer of autophagy, to some common chemotherapeutic drug regimens could increase cytotoxicity.

Several agents can cause autophagic cell death in different kinds of cancer, such as IFN-γ, resveratrol, vitamin D analogues, tamoxifen, arsenic trioxide, and actinomycin D. In glioma cell lines, it has been shown that arsenic trioxide can induce autophagy-related cell death by up-regulation of BNIP3 (a member of the Bcl-2 family), as well as its respective homologue BNIP3L. According to earlier findings, displacement of Beclin1 from its complex with Bcl-2 (BNIP3) enhances autophagy. Furthermore, BNIP3 over-expression also induced autophagy in some cell types [203]. Likewise, BNIP3 can make a key contribution to ceramide-mediated autophagy in malignant glioma cells [204]. An inorganic compound, sodium selenite, was also able to induce autophagy in malignant glioma cells via superoxide-induced mitochondrial damage [205]. Sodium selenite, ceramide and arsenic trioxide have all been shown to contribute to autophagic cell death by triggering mitochondrial damage. However, Δ^9^-tetrahydrocannabinol (THC) resulted in autophagy-related glioma cell death through induction of ER stress [206].

GBMs contain many hypoxic regions, and there can also be large necrotic regions inside the tumors. Increased expression levels of Bcl-2 family members leads to resistance to hypoxia in gliomas [207], and may result in increased resistance to some therapies. BH3 mimetics are small molecules, which can selectively attach to the BH3-binding groove of the anti-apoptotic Bcl-2 proteins. Therefore, BH3 mimetics are able to disturb the interaction between Bcl-2/beclin1 as well as between Bax/Bcl-2, in order to trigger autophagy or apoptosis in different kinds of cancer. In hypoxia-resistant malignant glioma cells, it was found that BH3 mimetics could induce autophagy-dependent cell death [207]. In addition, gossypol (a BH3 mimetic) specifically induces caspase-independent autophagic cell death [208].

p53 has been shown to be mutated in about one third of gliomas, and it can reduce the susceptibility of tumors to treatments that induce apoptosis [209]. Investigators have found that autophagy-related cell death can be induced in gliomas by addition of CQ, independently of the p53 status [210]. Nevertheless, p53 plays an essential role in governing autophagy in a variety of therapeutic approaches. Autophagy and DNA damage have both been induced by selective inhibitors of cyclooxygenase-2 (e.g. celecoxib) in glioma cells, which require a functional p53 pathway [211].

## MicroRNAs and autophagy in brain tumors

GBM is the most common as well as the most lethal primary tumor in the CNS [212, 213]. Nonetheless, all GBMs are not uniform and can display fundamental heterogeneity and may contain small sub-populations of cells, that have been called “glioma stem-like cells” (GSCs). One study suggested that GSCs are mainly responsible for tumor initiation, specify the malignant phenotype, cause therapy-resistance, and recurrence [214]. Analysis of gene expression has categorized GSCs extracted from patients into sub-types: mesenchymal (MES); classical (CL); and proneural (PN) [215–217]. Among them, MES GSCs have been suggested to be the most radiation-resistant and invasive cells [217]. The enhanced autophagic activities in the MES GSCs as compared to the PN GSCs, has been implicated in the high tumorigenicity and resistance to therapy [218].

MiR-93 is an important miRNA, which is highly expressed in different human cancers, and functions as one of the oncogenic miRNAs, through actuation of PI3K-AKT signaling pathways [219, 220]. Nevertheless, oncogenic effects of miR-93 has been considered to be depended on the contexts. As an instace, miR-93 acts as one of the tumour suppressors via suppressing parameters in a TGF-β signaling pathway, and the genes responsible for cell stemness such as *EZH1, SOX4, AKT3, STAT3, JAK1, CDKN1A,* and *CCND2* [221, 222].

Huang and colleagues examined two clinically relevant GBM subtypes, and found that miR-93 expression affected the GSC phenotype, together with the response to therapy, due to its effects on autophagy [223]. They also showed that miR-93 modulated autophagy functions in GSCs by synchronized suppression of several autophagy modulators, such as SQSTM1/p62, ATG4B, ATG5 and BECN1/beclin 1. Furthermore, two first-line GBM therapies, *Temozolomide* (TMZ) and irradiation (IR), as well as rapamycin (Rap) decreased the expression of miR-93, which itself, triggered the autophagic cascades in the GSCs. In fact, autophagy suppression using the ectopic expression of miR-93, or mediated by autophagy blockers, CQ and NSC (the ATG4B suppressor), increased TMZ as well as IR activities against the GSCs. The results suggested an important role for miR-93 in autophagy regulation, and proposed a combination therapeutic approach using autophagy suppression while administering cytotoxic treatment [223].

It has been shown that miR-30a has suppressive effects on autophagy, through direct targeting of beclin1 [224]. Xu and colleagues studied whether miR-30a enhanced TMZ cytotoxicity against GBMU251 cells, and the underlying mechanisms [225]. They found that TMZ therapy blocked the proliferation of U251 cells, while inducing apoptosis in a dose-dependent manner. Moreover, beclin1 and LC3-II expression levels, as well as the LC3-II to LC3-I ratio were significantly enhanced in TMZ-treated U251 cells in comparison to untreated cells. These results suggested that TMZ therapy could induce autophagy. Researchers found that TMZ therapy resulted in a considerable reduction of miR-30a expression levels in U251 cells in a dose-dependent manner. MiR-30a significantly suppressed autophagy induced by TMZ, as confirmed by the reduced levels of beclin1 and LC3-II, as well as lower ratio of LC3-II to the LC3-I, accompanied by elevated apoptosis as well as decreased proliferation of TMZ-treated U251 cells. Overall, this study showed that, miR-30a enhanced the TMZ chemosensitivity of GBMU251 cells through direct suppression of autophagy. Consequently, autophagy may be a target for improving the treatment effects against TMZ-resistant tumors [225].

Flavonoids (phenolic compounds derived from plants) have a wide range of pharmacological properties including antitumor activity. Studies have recently revealed the ability of flavonoids to affect cancer cell metastasis, angiogenesis, differentiation, proliferation, apoptosis, and multi-drug resistance [226]. The anticancer impact of luteolin (LUT), a naturally occurring flavonoid, includes suppression of metastasis, angiogenesis, cell proliferation and autophagy, as well as stimulation of apoptotic pathways [227]. During the passage through the intestinal mucosa, some LUT molecules are probably converted to glucuronides [228]. Due to its ability to cross the blood-brain barrier, LUT could be considered an appropriate molecule for the treatment of different brain tumors, such as GBM [229].

Ray and Chakrabarti showed that a combination of 50 μM SIL (silibilin, a flavonolignan) and 20 μM LUT synergistically inhibited the growth of T98G and GBMU87MG cells, and the combination of these two natural compounds was more effective than conventional chemotherapy (100 μM TMZ or 10 μM BCNU) [230]. The SIL and LUT combination suppressed GBM cell growth via inducing apoptosis and inhibiting tumor cell migration and invasion. Additionally, the SIL and LUT combination repressed rapamycin (RAPA)-mediated autophagy by PKCα suppression, and promoted apoptosis through iNOS down-regulation. The combination also significantly enhanced the expression of the tumor inhibitor miR-7-1-3p in GBM cells. It was also shown that miR-7-1-3p over-expression increased the antitumor activity of SIL and LUT in RAPA-pre-treated T98G and U87MG cells. Consequently, these findings indicated that miR-7-1-3p over-expression enhanced the antitumor effects of SIL and LUT to induce apoptosis and suppress autophagy in several human GBM-cells, both in-vivo and in-vitro [230].

Under hypoxic conditions, autophagy can have a protective effect on cancer cells. Moreover, hypoxia affects protein stability, mRNA stability, and also mRNA transcription. In addition, hypoxia causes a shift in expression levels of a particular class of miRNAs [231]. The mechanisms of miRNA-associated hypoxia-induced autophagy in GBM are not yet fully understood. A study using miRNA microarray analysis in GBM cells, revealed the differential expression of several miRNAs under hypoxic condition [232]. It has also been reported that miR224-3p could be implicated in the regulation of hypoxia-mediated autophagy in GBM cells. The over-expression of miR224-3p inhibited hypoxia-induced autophagy, while its down-regulation promoted autophagy under normoxic conditions. Moreover, one study [232] reported that miR224-3p directly suppressed the expression of two autophagy-associated genes i.e., ATG5 & FAK family interacting protein of 200 kDa (FIP200) and therefore inhibited autophagy. Furthermore, miR224-3p enhanced hypoxia-induced apoptosis and decreased cell proliferation in vitro, and its over-expression inhibited GBM tumorigenesis in vivo. These authors demonstrated that miR224-3p is a new down-regulated miRNA in hypoxia, and could be a significant autophagy regulator by suppression of ATGs in GBM cells.

Vestibular schwannoma (VS) is a Schwann cell tumor of the vestibular nerves, and comprises about 10% of intracranial neoplasms [233]. VS occurs in both familial (neurofibromatosis type 2, *NF2*) and sporadic forms, both of which are related to defects in the *NF2* gene [233]. In one investigation, the antitumor effects of ailanthone (AIL) (a quassinoid compound derived from the traditional Chinese medicinal herb, *Ailanthus altissima*) against VS was evaluated by Yang and colleagues [234]. Different doses of AIL (0–1 μM) were used to treat VS cells, and autophagy, apoptosis, cell viability and proliferation were evaluated. After miRNA transfection, miR-21 expression was increased in VS cells. AIL significantly decreased VS cell viability. In response to 0.6 μM AIL, p62 was down-regulated, beclin-1 and LC3-II were accumulated, caspase 3 and caspase-9 were cleaved, the rate of apoptotic cells was increased, and the expression of cyclin D1 as well as the proportion of BrdU^+^ cells were decreased. miR-21 was poorly expressed in cells treated with AIL, and furthermore AIL-mediated autophagy and apoptosis were reduced by the over-expression of miR-21. Furthermore, AIL down-regulated Raf and Ras, and also deactivated p70S6K, mTOR, ERK and MEK, whereas the deactivation and down-regulation of these mediators induced by AIL were reversed by miR-21 over-expression. Consequently, AIL suppressed the proliferation of VS cells and induced autophagy and apoptosis. The anticancer properties of AIL in VS cells were explained by miR-21 down-regulation and consequent suppression of the mTOR and Ras/Raf/MEK/ERK pathways [234].

Approximately, 30% of patients with medulloblastoma, another malignant pediatric brain tumor [235], undergo metastasis at an early stage, and therefore they have a poor prognosis. If harsh cytotoxic therapy is administered to children whose brain is still developing, the surviving patients can suffer long-lasting developmental, endocrine and neurocognitive deficits. Therefore, in order to achieve a more effective treatment for medulloblastoma with fewer side effects, it is essential to discover more targeted therapeutic approaches based on validated biological mechanisms. It is believed that, because the SHH and WNT developmental pathways are involved, medulloblastoma could result from the deregulated expansion of cells in the nervous system [235]. Moreover, one study carried out by Singh et al. found low expression levels of miR-30a (which targets beclin1) in the medulloblastoma cell lines, D425, D283, and Daoy. Restoring the miR-30a expression level blocked tumorigenicity, and reduced the clonogenic potential as well as the proliferation of the medulloblastoma cells. It was proposed that miR-30a down-regulates the expression of beclin1, and suppresses autophagy in medulloblastoma cell-lines, by LC3B down-regulation. This could be reversed by CQ therapy, which induces starvation-induced autophagy. Therefore, miR-30a could be considered as a treatment approach for medulloblastoma, via suppressing autophagy, reversing the malignant phenotype, and reducing survival of cancer cells [236].

Table 2 lists some of the various miRNAs that have been associated with autophagy in brain tumors.

**Table 2:** Selected autophagy-related miRNAs in brain tumors

Brain tumor	miRNA	Expression	Effect (s) on autophagy	Target (s)	Study outcome (s)	Model	Cell line	Ref
GBM	miR-93	Up	Inhibit autophagy	BECN1/Beclin 1, ATG5, ATG4B, SQSTM1/p62	Autophagy inhibition increased antitumor effects of Rap, IR, and TMZ on glioma stem-like cells	In vitro, In vivo	U87	[237]
	miR-30a	Up	Inhibit autophagy	Beclin 1	MiR-30a over-expression increased the cytotoxicity of TMZ to U251 cells	In vitro	U251	[225]
	miR-224-3p	Up	Inhibit autophagy	ATG5	Mir-224-3p suppressed metastasis. It also enhanced the chemo-sensitivity of LN229 cells in hypoxic conditions through autophagy suppression	In vitro, In vivo	LN229	[238]
	miR-17	Up	Inhibit autophagy	ATG7	The activation of autophagy by anti-miR-17 led to a decrease of the threshold resistance at temozolomide doses in T98G cells. Modulation of miR-17 led to sensitization to low dose ionizing radiation in U373-MG cells	In vitro	T98G, U373-MG	[115]
	miR-340	Up	Inhibit autophagy	XIAP, BMI1, ROCK1	MiR-340 reduced cell growth, inhibited cell motility, and regulated glioma development	In vitro	U87MG, U251MG, U373, A172, U118, T98G, SHU-44	[239]
	miR-224-3p	Up	Inhibit autophagy	ATG5, FIP200	MiR224-3p increased hypoxia-induced apoptosis, inhibited hypoxia-induced autophagy, reduced cell proliferation in vitro, inhibited tumorigenesis of GBM cells in vivo	In vitro, In vivo	U251, U87	[232]
	miR-517c	Up	Inhibit autophagy	Tp53	Mir-517c suppressed autophagy and decreased tumor invasion	In vitro, In vivo	U251, U87	[240]
	miR-7-1-3p	Up	Inhibit autophagy	PKCa, mTOR, SQSTM1, p62, XIAP	MiR-7-1-3p over-expression potentiated silibinin & luteolin to induce apoptosis and inhibit autophagy	In vitro, in vivo	U87MG, T98G	[230]
	miR-138	Up	Inhibit autophagy	LC3-II, BIM	MiR-138/BIM axis regulated autophagy-mediated resistance to TMZ	In vitro, In vivo	LN-18, LN-229, LN-308, LN-319, LN-428, D247MG, A172, U87MG, T98G	[241]
	miR-155-3p	Up	Activate autophagy	LC3B-II, SQSTM1	MIR155-3p enhanced hypoxia-induced autophagy through targeting the CREBRF-CREB3-ATG5 pathways	In vitro, In vivo	U251, T98G	[242]
	miR-30e	Up	Inhibit autophagy	Beclin-1	Combination of proanthocyanidin and miR-30e suppressed sodium sulfite-induced autophagy	In vitro	GSC, SNB19	[243]
	miR-128	Up	Activate autophagy	mTOR, RICTOR, IGF1, PIK3R1	MiR-128 directly blocked mTOR pathway and induced glioma cell death	In vitro	Hs683, M059K, U87MG	[244]
	miR-590-3p	Up	Activate autophagy	LC3-II, Beclin-1,	TMZ combined with endothelial-monocyte actuating polypeptide II inhibited malignant phenotype of GSCs through miR-590-3p/MACC1 suppressing the PI3K/AKT/mTOR signaling pathways	In vitro, In vivo	U87, U251	[245]
Scwannoma	miR-21	Up	Inhibit autophagy	LC3-II, Beclin-1	Ailanthone-induced autophagy & apoptosis, suppressed proliferation of vestibular schwannoma cells	In vitro	vestibular schwannoma	[234]
	miR-210	Up	Activate autophagy	P62, elf4E	Inhibition of miR-210 promoted tumor cell apoptosis and cell cycle arrest, decreased angiogenesis, and activated autophagy	In vitro	RT4-D6P2T	[246]
Medulloblastoma	miR-30a	Up	Inhibit autophagy	LC3B, Beclin-1	Mir-30a inhibited tumorigenicity and growth of medulloblastoma cell lines, and suppressed autophagy	In vitro, In vivo	Daoy, D283, D425	[236]
	Let-7f-1	Up	Inhibit autophagy	HMGB1	SPARC regulated cisplatin resistance by regulating the Let-7f-1 miRNA/HMGB1 axis	In vitro, In vivo	D425 UW228	[247]
Glioma	miR-193a-5p	Up	Activate autophagy	LCII/LCI, Beclin-1	CASC2 is down-regulated in glioma, leading to enhanced levels of miR-193a-5p and decreased expression of mTOR, resulting in increased autophagy	In vitro	U257, U87	[248]
Astrocytoma	miR-224-3p	Up	Inhibit autophagy	ATG5	HIF-1α/miR-224-3p/ATG5 axis influenced chemosensitivity and cell mobility through modulating hypoxia-mediated autophagy	In vitro, In vivo	U-251MG	[245]

## Conclusion

Due to the generally poor survival of patients diagnosed with malignant brain tumors (especially GBM), it is necessary to discover novel therapeutic strategies with fewer side effects. According to mounting evidence, disturbed autophagy critically contributes to the pathogenesis as well as progression of brain tumors. Therefore, new rationally designed drugs are needed, that should be soundly based on the underlying mechanisms of autophagy. Recent data shows that miRNAs can regulate and influence autophagy through different pathways. Therefore, miRNAs such as miR-30a, could be considered as a new therapeutic approach for the therapy of brain tumors, via suppressing autophagy, which has been shown to play a role on the malignant phenotype, survival and growth of cancer cells. The present review has summarized studies related to this concept, but it is obvious that there is still a long way to go before miRNA-based drugs could be used for brain tumor treatment. In order to find novel potential drugs for brain tumors, further attention should be focused on the regulatory properties of different miRNAs in the autophagy cascade. To reach this goal, more experimental studies must be conducted to clarify the underlying molecular mechanisms, and then clinical trials could be warranted to prove the effectiveness and safety of therapies based on miRNAs.

## Abbreviations

AGO: Argonaute
ATG: Autophagy-related *gene*
AMAPK: AMP-activated protein kinase
*BECN1*: Beclin 1
BNIP3: Bcl-2 nineteen-kilodalton interacting protein 3
CAMKK2: Calcium/calmodulin-dependent protein kinase kinase 2
CDKN1A: Cyclin Dependent Kinase Inhibitor 1A
CCND2: Cyclin D2
CL: Classical
CNS: Nervous system
CSF: *Cerebrospinal fluid*
DRAM2: DNA damage-regulated autophagy modulator 2
CQ: Chloroquine
EGFR: *Epidermal growth factor receptor*
ERK: Extracellular Signal-regulated Kinase
EXH1: Enhancer of zeste homolog 1
GBM: Glioblastoma
HCC: GABARAPL1: GABA Type A Receptor Associated Protein Like 1; Hepatocellular carcinoma
*LAMP-1*: Lysosomal-associated membrane protein 1
LC3: Light chain 3
LncRNA: Long non-coding RNA
LUT: Luteolin
MEFs: Mouse embryonic fibroblasts
MEK: Mitogen-activated protein kinase
MES: Mesenchymal
MREs: miRNA response elements
mTORC1: Mammalian target of rapamycin complex 1
NF1: Neurofibromatosis type I
HOPS: Homotypic fusion and protein sorting-tethering complex
PKM1: Pyruvate kinase isozymes M1
PTB1: Polypyrimidine tract-binding protein 1
PE: Phosphatidylethanolamine
PDGFR: *Platelet-derived growth factor receptors*
PI3K: Phosphatidylinositol 3-kinase
*PIK3C3*: Phosphatidylinositol 3-Kinase Catalytic Subunit Type 3
PML: Promyelocytic leukemia
PN: Proneural
PHLPP: PH domain and leucine rich repeat protein phosphatase 1
PTEN: Phosphatase and tensin homolog
RCC: Renal clear cell carcinoma
RISC: RNA-induced silencing complex
RAB1B: Ras-related protein Rab-1B
TTKs: *Receptor tyrosine kinases*
RUNX3: Runt-related transcription factor 3
SQSTM1: Sequestosome-1
SIRT1: Silent mating type information regulation 2 homolog 1
SNAREs: Soluble N-ethylmaleimide-sensitive agent attachment protein receptors
SNAP29: Synaptosomal-associated protein 29
STX17: Syntaxin 17
STAT3: *Signal transducer and activator of transcription 3*
TIGAR: TP53-inducible glycolysis and apoptosis regulator
TKI: Tyrosine kinase inhibitors
THC: Tetrahydrocannabinol
TRPM3: Transient receptor potential melastatin 3
VAMP8: Vesicle-associated membrane protein 8
VHL: Von Hippel–Lindau
VPS34: Vacuolar protein sorting 3
VS: Vestibular schwannoma
ULK: Unc-51-like kinase
YY1: Yin Yang 1a
